# Diverse regulation of plasmodesmal architecture facilitates adaptation to phloem translocation

**DOI:** 10.1093/jxb/erz567

**Published:** 2019-12-24

**Authors:** Dawei Yan, Yao Liu

**Affiliations:** 1 State Key Laboratory of Crop Stress Adaptation and Improvement, State Key Laboratory of Cotton Biology, School of Life Sciences, Henan University, Kaifeng, China; 2 University of Essex, UK

**Keywords:** Callose, phloem translocation, plasmodesmata, symplastic trafficking, unloading

## Abstract

The long-distance translocation of nutrients and mobile molecules between different terminals is necessary for plant growth and development. Plasmodesmata-mediated symplastic trafficking plays an important role in accomplishing this task. To facilitate intercellular transport, plants have evolved diverse plasmodesmata with distinct internal architecture at different cell–cell interfaces along the trafficking route. Correspondingly, different underlying mechanisms for regulating plasmodesmal structures have been gradually revealed. In this review, we highlight recent studies on various plasmodesmal architectures, as well as relevant regulators of their *de novo* formation and transition, responsible for phloem loading, transport, and unloading specifically. We also discuss the interesting but unaddressed questions relating to, and potential studies on, the adaptation of functional plasmodesmal structures.

## Teaser

A review of recent studies on plasmodesmal architecture and relevant regulators of their formation and transition involved in phloem translocation.

## Introduction

Phloem-mediated translocation of photoassimilates, such as sucrose, is one of the most critical processes for plant growth and development. It includes three steps: loading, transport, and unloading. The supply end that produces and exports assimilates is termed the ‘source’, while the consumption end is referred to as the ‘sink’. From source to sink, both symplastic and apoplastic trafficking are involved, and their contributions vary in different organs/tissues and species (Comtet *et al.*, 2017; Milne *et al.*, 2018). In this review, we will focus on symplastic trafficking in higher plants.

Plant cells, unlike animal cells, are isolated to some extent by their surrounding cell wall. To facilitate intercellular communication, plants have formed specialized plasma membrane-lined tubes, termed plasmodesmata (PD), that connect neighboring cells to form a continuous multicellular cytoplasm known as the symplast (Faulkner, 2018). The symplast allows both local and long-distance molecular transport through the phloem (Ayre *et al.*, 2003). To date, many molecular species are known to traffic through the PD and are translocated by the phloem, including nutrients, proteins, RNAs and hormones, indicating that they may function in expanded domains (Turgeon and Wolf, 2009; Ham and Lucas, 2017). Furthermore, symplastic trafficking is crucial and contributes to plant development and stress responses by transporting mobile signaling molecules, such as ABA and salicylic acid (Wang *et al.*, 2013; Yadav *et al.*, 2014; Lim *et al.*, 2016; Otero *et al.*, 2016; Li *et al.*, 2018; Tylewicz *et al.*, 2018).

**Fig. 1.**
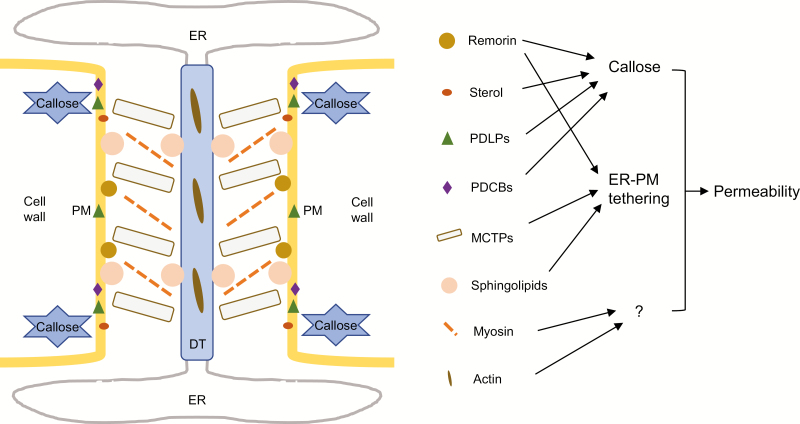
A schematic diagram showing the major localized plasmodesmal components that are involved in permeability control. These components are localized at the plasmodesma (left panel, except choline) and modulate plasmodesmal permeability by different mechanisms (right panel). Remorin, sterol, PDLPs, and PDCBs can induce callose biosynthesis and thus restrict plasmodesmal aperture. Remorin, MCTPs and sphingolipids affect the ER–plasma membrane connection and control the cytoplasmic sleeve space. Myosin and actin were previously reported, without a detailed working model, to alter the plasmodesmal permeability. DT, desmotubule; ER, endoplasmic reticulum; MCTP, multiple C2 domains and transmembrane region protein; PDCB, PD CALLOSE-BINDING PROTEIN; PDLP, PD-LOCALIZED PROTEIN; PM, plasma membrane.

Typical PD consist of the outer plasma membrane and appressed endoplasmic reticulum (ER), termed the desmotubule, along with the supporting cell wall, creating the foundations for diverse plasmodesmal structures (Tilsner *et al.*, 2016) (Fig. 1). PD are classified as simple and branched based on their morphologies. The simple form can be transformed into the branched form during sink–source transition in leaves, accompanied by reduced conductivity for macromolecules (Oparka *et al.*, 1999). Recently, a separate classification that differentiates types I and II PD has been developed, which is dependent on the status of the cytoplasmic sleeve between the plasma membrane and desmotubule (Nicolas *et al.*, 2017). Type I PD lack visible cytoplasmic sleeve and internal tethers, but are surprisingly more permeable to molecules than type II PD, which possess a visible sleeve (Yan *et al.*, 2019).

Callose, an extracellular polysaccharide deposited around the PD (Fig. 1), is a well-known regulator of plasmodesmal aperture and the permeability of the corresponding cell-to-cell interface (Zavaliev *et al.*, 2011; De Storme and Geelen, 2014; Cui and Lee, 2016). Although other factors, including reactive oxygen species (Benitez-Alfonso and Jackson, 2009), PD-localized proteins (Simpson *et al.*, 2009; Lee *et al.*, 2011), and sterol (Grison *et al.*, 2015) (Fig. 1), have been reported to control plasmodesmal permeability, their downstream terminal targets are callose. A previous study found that salicylic acid signaling triggered plasmodesmal closure by altering lipid organization in a manner dependent on the protein remorin. Moreover, callose levels increased on both the apical/basal and lateral walls of salicylic acid-treated root cells (Huang *et al.*, 2019), implying combined modulation of plasmodesmal gating. Unlike these cases, in *phloem unloading modulator* (*plm*) mutants, a reduction in plant complex sphingolipids with saturated very-long-chain fatty acids (VLCFAs) led to defective plasmodesmal ultrastructure maintenance without altering callose accumulation (Yan *et al.*, 2019), a case that will be described in detail below. Another example of callose-independent dynamic control of plasmodesmal transport by light and the circadian clock has been recently revealed; however, the direct regulation mechanism was not uncovered (Brunkard and Zambryski, 2019).

Additionally, more novel models of plasmodesmal permeability modulation have been proposed. Mechanosensitive control indicates that the pressure forces resulting from changes in the environment disturb the equilibrium position of the ER–desmotubule complex, as well as inducing plasmodesmal closure and transport blocking (Park *et al.*, 2019). Along with previous work on plasmodesmal ultrastructures (Nicolas *et al.*, 2017; Brault *et al.*, 2019), the selective role of tethers connecting ER to plasma membrane in plasmodesmal trafficking is highlighted in Fig. 1. INCREASED SIZE EXCLUSION LIMIT (ISE)1 and ISE2 are two chloroplastic DEVH-BOX RNA helicases that were found to positively control secondary complex PD formation in embryos and adult leaves via an elusive mechanism (Kobayashi *et al.*, 2007; Burch-Smith and Zambryski, 2010), establishing a link between chloroplast and plasmodesmal function. Moreover, the cytoskeleton (Hsieh *et al.*, 2017; Diao *et al.*, 2018), myosin (Radford and White, 2011) (Fig. 1), and cell wall formation (Knox and Benitez-Alfonso, 2014) were all thought to potentially influence plasmodesmal permeability. However, direct evidence indicating alterations of plasmodesmal internal organization is still lacking.

In this review, we summarize the diverse forms of PD and architecture regulation at various cell-to-cell interfaces that contribute to phloem loading, transport, and unloading processes, as well as the relevant techniques for studying plasmodesmal ultrastructure. Additionally, we discuss unaddressed questions and potential approaches for future investigations on the regulatory mechanisms of plasmodesmal structures and functions.

### Regulation of the conductivity of pore-plasmodesmata units that connect companion cells and sieve elements modulates phloem loading

**Fig. 2.**
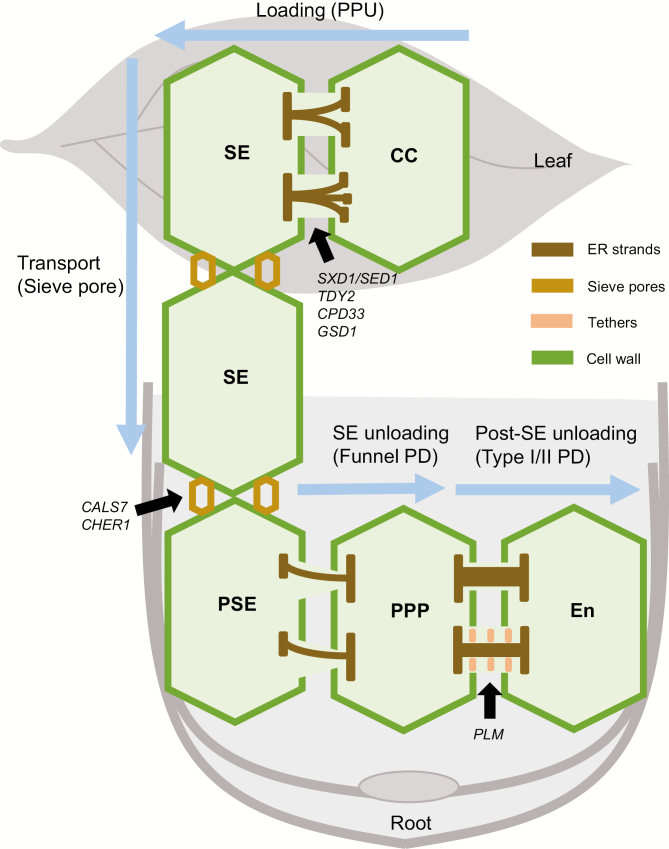
Diverse plasmodesmal forms and regulators responsible for phloem translocation. In source tissues, mobile molecules that are present in CCs diffuse through PPUs into SEs, followed by long-distance transport in SE cells that are connected by sieve pores within sieve plates. At the region of differentiated PSE, those molecules are batch-unloaded into PPP via funnel-shaped PD, and subsequently moved further into the endodermis. The post-SE unloading process through the PPP–endodermis interface can be modulated by the dynamic proportion of type I and type II PD. The key genes that play regulatory roles are listed. CC, companion cell; En, endodermis; ER, endoplasmic reticulum; PPP, phloem pole pericycle; PPU, pore-PD unit; SE, sieve element.

The phloem consists of conducting cells, namely sieve elements (SEs), and their surrounding companion cells (CCs) and parenchyma (Knoblauch and Oparka, 2012; Heo *et al.*, 2014). After asymmetric division from the same precursor cell as the CC, an SE undergoes a maturation process, similar to programmed cell death with enucleation and the loss of some organelles (Furuta *et al.*, 2014). Eventually, only partial cellular components are retained, including plastids, mitochondria, P-proteins, and the smooth ER structure (Oparka and Turgeon, 1999). Therefore, mature SEs must rely on CCs to survive by the aid of remodeled pore-PD units (PPUs) that connect SEs and CCs to form a complex system (Lee and Frank, 2018) (Fig. 2). Each PPU consists of hundreds of branched plasmodesmal strands on the CC side and a wide pore on the SE side. This asymmetric shape satisfies the high-volume load from the CC to the SE side (Oparka and Turgeon, 1999). Two pathways are thought to be responsible for trafficking via PD: the cytoplasm and the ER/desmotubule. SEs, however, have no integral cytoplasm in which the desmotubule connecting the residual ER and the normal CC ER may provide a potential channel for flow (Blackman *et al.*, 1998; Oparka and Cruz, 2000; Heo *et al.*, 2017).

Another notable property of PPUs is their size exclusion limit, which is up to 70 kDa (Oparka and Turgeon, 1999) and is likely required for the loading of larger macromolecules. Thus, the reduction of plasmodesmal permeability would result in compromised phloem loading. For example, in maize, three mutants were reported to show defects in the symplastic transport of photoassimilates, including *sucrose export defective1* (*sxd1/sed1*), *tie-dyed2* (*tdy2*), and *Carbohydrate partitioning defective33* (*Cpd33*). The *sxd1* mutation resulted in a tocopherol deficiency and overproduced callose at the bundle sheath–vascular parenchyma interface that led to abnormal plasmodesmal morphology and prevented symplastic transport in leaf blades (Russin *et al.*, 1996; Botha *et al.*, 2000). Because tocopherol is an antioxidant that protects chloroplasts from photooxidative damage, callose deposition may be the consequence of enhanced oxidative stress in the source leaves of the *sxd1* mutant (Hofius *et al.*, 2004). *TIE-DYED2* (*TDY2*), a callose synthase gene, is highly expressed in the vascular tissues of developing leaves. Loss-of-function does not affect either callose accumulation or the plasmodesmal structure and frequency but does affect sucrose movement between CCs and SEs, as well as vascular differentiation (Baker and Braun, 2008; Slewinski *et al.*, 2012). Like *TDY2*, the absence of *CPD33*, which encodes multiple C2 domains and transmembrane region proteins (MCTPs), resulted in a reduced number of PD at the CC–SE interface, with consequently weakened sucrose export (Tran *et al.*, 2019). In rice, overexpression of *GRAIN SETTING DEFECT1* (*GSD1*), which encodes a putative remorin protein with specific expression in CCs, inhibited plasmodesmal permeability and the export and translocation of soluble sugars by increasing callose deposition (Gui *et al.*, 2014).

Although the unique structures of PPUs have been discovered over the years, how these PPUs are formed during plant developmental phases has not been well studied. As phloem loading does not usually occur in sink tissues, it would be interesting to isolate the PD in the same leaf undergoing a sink–source transition and perform morphological, transcriptomic, and proteomic analyses. However, optimization of the purification method is required.

### Sieve-plate pores deformed from plasmodesmata facilitate long-distance transport

As conducting cells, SEs are the most highly specialized cells in the phloem. Individual SEs are connected one by one to establish a long semi-hollow tube, with sieve pores formed in the sieve plates facilitating mass flow inside (Fig. 2). Sieve pores are differentiated from the original PD during SE maturation (Kalmbach and Helariutta, 2019). At the beginning of the transformation, callose is deposited around the edges and replaces cellulose in the primary cell wall (Esau and Thorsch, 1985). When a sufficient amount of callose has filled the whole area of the future pore, the degradation of callose commences until a larger pore of up to 1 µm is formed; callose is retained only around the pore (Esau and Cheadle, 1959; Esau and Thorsch, 1985; Mullendore *et al.*, 2010).

Due to its essential contributions, callose has been found to be involved in the modulation of the sieve pore diameter. In mutants of *callose synthase 7* (*cals7*), a gene with specific expression patterns in SEs, significant depletion of callose deposition was observed at the sieve plate, which led to reduced density, smaller sieve pores, and impaired root growth (Barratt *et al.*, 2011; Xie *et al.*, 2011). With the loss of its central components after maturation, the sieve pore is no longer a real or complete plasmodesma, and regulation of plasmodesmal permeability, depending on the membrane microdomain, is not applicable. However, callose still serves as a dynamic regulator. For instance, in response to local phloem damage, callose is instantly deposited extracellularly around the pores and constricts the sieve pore corridor. The Ca^2+^ concentration is the stimulus for this process (Amsbury *et al*., 2017). Additionally, overproduction of callose in the phloem by *cals3m*, a gain-of-function mutation of *CALS3*, inversely blocks SE transport (Vatén *et al*., 2011).

SE differentiation and enucleation are governed by the NAC DOMAIN CONTAINING PROTEIN (NAC) 45/86-DEPENDENT EXONUCLEASE-DOMAIN PROTEINS (NENs) pathway. The double mutant *nac45/86* exhibits the formation of sieve pores but defective phloem transport and unloading (Furuta *et al.*, 2014), suggesting the potential indirect control of sieve pore development by SE enucleation. The identification of *CHOLINE TRANSPORTER-LIKE 1* (*cher1*) mutants provided evidence for the link between sieve pore biogenesis and lipid homeostasis, as well as the presence of PD-localized proteins (Kraner *et al.*, 2017*b*). These *cher1* mutants have fewer, smaller, and structurally abnormal sieve pores in their roots, resulting in impaired phloem translocation (Dettmer *et al.*, 2014). Another study found that plasmodesmal maturation from simple to branched PD was suppressed in *cher1* leaves, leading to the absence of complex PD (Kraner *et al.*, 2017*a*). Thus, these two structural reconstructions may be similar to some extent and may involve common regulatory factors. However, which lipid components are responsible for the sieve pore and plasmodesmal defects has yet to be determined, as the supply of choline did not complement the *cher1* mutant, and the difference in global lipid composition was not dramatic (Kraner *et al.*, 2017*b*). Thus, it is highly possible that other proteins or local lipid species, apart from choline, are potential cargos of CHER1. Comparative proteomic profiling of PD in Arabidopsis wild-type Col-0 and *cher1* leaves identified plasmodesmal structure-associated proteins that were already known but also some that were newly discovered (Kraner *et al.*, 2017*b*). These promising candidates include C2 calcium/lipid-binding plant phosphoribosyl transferase and maternal effect embryo arrest protein (MEE9), which were both localized in the PD (Kraner *et al.*, 2017*b*).

### Specific funnel-plasmodesmata mediate sieve element unloading with unknown regulation mechanism

The SE unloading domain in root tips was first revealed by green fluorescent protein (GFP) fusion proteins expressed under the CC-specific *AtSUC2* promoter. Unlike free GFP, all soluble GFP-fusion variants were unloaded but were restricted to a narrow zone of cells adjacent to the mature protophloem (Stadler *et al.*, 2005). This region, which is responsible for protophloem sieve element (PSE) unloading, was ~300 µm long starting from the first differentiating protophloem cell (Ross-Elliott *et al.*, 2017). The adjacent cells that connect PSE, called phloem pole pericycle (PPP), act as the principle contributors to SE unloading. This role was further confirmed by the blocked PSE–PPP movement after plasmodesmal closure as a result of specific induction of callose accumulation around the SE–PPP interface using the inducible *cals3m* system. Beyond this unloading zone toward the mature part of the root, callose was strongly accumulated at the SE cell walls, which reduced external flow by blocking the corresponding PD (Ross-Elliott *et al.*, 2017).

Interestingly, it was found that molecular unloading does not occur at a constant rate but in distinct pulses, termed ‘batch unloading’ (Ross-Elliott *et al.*, 2017), suggesting unusual properties of the PD in this interface. Indeed, a novel architecture, termed ‘funnel PD’, was observed solely at the PSE–PPP interface (Ross-Elliott *et al.*, 2017). Asymmetric funnel-shaped PD exhibit larger apertures toward the PSE entrance but narrow toward the PPP side, enabling bulk flow for unloading with low pressure (Fig. 2). Additionally, the relative distribution and total number of PD between the PSE–PPP interface are both higher than that at the PSE–CC and PSE–metaphloem interfaces in the unloading zone. However, ~10% of normal simple PD were found at the PSE–PPP junction (Ross-Elliott *et al.*, 2017). Due to the small proportion, their contributions to SE unloading may be minor.

Until recently, the formation and regulation of funnel-PD development were elusive in the absence of identification or characterization of relevant mutants. It may be worth investigating the genes that affect plasmodesmal complexity, such as *CHER1*, which controls the formation of complex PD. Due to the universal role of callose in plasmodesmal gate control, the absence of CALS may functionally impair funnel PD. Among the 12 CALS members in Arabidopsis, CALS7 (Xie *et al.*, 2011) and CALS8 (Yan *et al.*, 2019) exhibit specific expression patterns in SEs and PPP, respectively, and may be excellent candidates.

### Sphingolipids maintain the plasmodesmal ultrastructure specifically for post-sieve element unloading

The PPP acts as a repository for unloaded macromolecules and is a potential filter in root tip cells for molecules that are subsequently transported beyond the PPP to their destination. Trafficking from PPP to its neighboring cell layer, the endodermis, is termed ‘post-SE unloading’ (Patrick, 1997; Yan *et al.*, 2019) (Fig. 2). While the SE–PPP interface is designed to promote SE unloading, the PPP–endodermis interface is the inverse bottleneck for the post-SE unloading process. All simple, branched, type I and type II PD, including a few intermediates with no clear spokes and partial detachment between the ER and plasma membrane, were observed at the PPP–endodermis interface (Yan *et al.*, 2019). Unlike the equal percentages of type I and type II, simple PD are more numerous than branched PD, which is consistent with the observation that simple PD are more permeable. Interestingly, our previous study found that the *plm* mutant lacked the typical type II PD. This absence of type II PD, however, is specific to the PPP–endodermis interface, but not the SE–PPP interface, in the roots (Yan *et al.*, 2019), which indicates a unique functional property of PLM for post-SE unloading. This information, therefore, reinforces the distinguishable difference between the SE and post-phloem transport.

Consistent with the near loss of the sleeve, no significant differences were detected between the width of simple and branched PD in the *plm* mutant. Additionally, the ratio of simple and branched PD was similar to that in the wild-type (Yan *et al.*, 2019), implying potential independent mechanisms for the modulation of these two morphological transformations.

Type I PD were enriched in young tissues with newly divided cell walls, while type II PD were more numerous in relatively older tissues. Unexpectedly, the extreme narrow sleeve of type I PD enabled also small molecule diffusion and macromolecule trafficking (Nicolas *et al.*, 2017). More surprisingly, the predominant presentation of type I PD at the PPP–endodermis interface increased its conductivity, leading to enhanced post-phloem unloading and earlier root elongation (Yan *et al.*, 2019). These findings challenge the previous model, which assumes that the ER–plasma membrane gap is the space for the transport stream (Epel, 1994; Kragler *et al.*, 1998), suggesting the need to reconsider the accessible route through the PD. A supporting case is the observation of fluorescent probe trafficking between cells via the desmotubule in tobacco and *Torenia* (Cantrill *et al.*, 1999). Recently, MCTPs were found to act as ER–plasma membrane tethers of the PD (Brault *et al.*, 2019) (Fig. 1), which may regulate the ER–plasma membrane contacts within the PD. This work uncovers part of the mysterious internal structure of the PD and directs us to reconsider the role of these tethers in internal molecular transport. Specifically, is this role positive, negative, or both? Future investigations on the diverse selective and non-selective trafficking routes within the plasmodesmal channel and plasmodesmal structural modification would allow us to understand the evolution and adaptation of the PD in changing developmental contexts.

Characterization of the *PLM* gene revealed a mechanism of specific membrane components for maintaining the plasmodesmal structure and function with no effect on plasmodesmal density and callose deposition. *PLM* encodes a novel protein involved in the biosynthesis pathways of VLCFA-containing sphingolipids, especially ceramides and glycosyl inositol phosphoryl ceramides (GIPCs) (Yan *et al.*, 2019). Based on the enrichment of GIPCs in plasmodesmal microdomains (Grison *et al.*, 2015) (Fig. 1), we speculate that this deficiency may influence the maintenance of the plasmodesmal architecture to some extent. However, a link between ceramides and PD has been introduced. Their interaction may be either direct (i.e. regulation of plasmodesmal functions by ceramides) or indirect (i.e. ceramides act as the backbone for the GIPCs or as signal molecules for plasmodesmal modification). It has been demonstrated that inhibition of ceramide synthesis affects the endomembrane ultrastructure and that VLCFA-sphingolipids are involved in the molecular secretory pathway (Markham *et al.*, 2011). Therefore, various loss-of-function mutants of sphingolipid biosynthesis genes are excellent tools for further clarifying the relationship between plant sphingolipids and plasmodesmal structural organization.

### Updated approaches for observation of plasmodesmal structure

As nanoscale channels (diameter of 20–50 nm) embedded in the cell walls between two adjacent cells, PD are difficult to observe by conventional light microscopy. Therefore, plasmodesmal research is highly dependent on technical developments. Electron microscopy (EM) was first developed in 1931 and became a common technique in the 1950s. Due to the shorter wavelength of electrons than photons, EM provides images at much higher resolution, up to 1 nm. This advancement enhanced the study of plasmodesmal ultrastructure and laid the foundation for current models of plasmodesmal architecture (Roberts, 2018). However, one problem of this method is that the chemical fixation or embedding procedure may cause structural artifacts due to poor sample penetration and slow cellular matrix crosslinking (Gilkey and Staehelin, 1986). With the development of cryo-EM, which uses rapid low-temperature fixation and high-pressure freezing, the cellular architecture was preserved, and the native plasmodesmal structure was revealed (McIntosh *et al.*, 2005).

However, as conventional EM is two-dimensional, information on the whole plasmodesmal internal architecture is still missing. Recently, a new electron tomography method has provided excellent images displaying the three-dimensional organization of PD. Technological advancements include the acquisition of tilt series of longitudinal views ranging from −70° to +70° at high magnifications and subsequent tomogram reconstruction, combination, and image segmentation (Nicolas *et al.*, 2017). By this method, two types of PD were clearly presented with evident differences (described above).

Additionally, field emission scanning electron microscopy (FESEM) can be used to image PD while they are exposed on the sample surface. Thus, cryo-FESEM, which uses similar pre-preparation methods as cryo-EM, has been exploited to reveal the plasmodesmal details within the cell wall (Vesk *et al.*, 2000). An example of this feat is the remarkably clear images of sieve plates and sieve pores acquired from a range of species obtained by Faulkner *et al.* (2008) and Mullendore *et al.* (2010).

Meanwhile, new advances in fluorescence imaging using confocal laser scanning microscopy have been widely used for fluorescent molecules and structures. However, due to the diffraction limit of light (~200 nm) and insufficient labeling markers for the plasmodesmal microdomain and its components, the capacity for resolving fine details of plasmodesmal ultrastructure with high resolution is unsatisfactory. Improvements in super-resolution imaging surpassed the diffraction barrier and bridged the gap between fluorescence and electron microscopy (Huang *et al.*, 2009; Lippincott-Schwartz and Patterson, 2009; Huang, 2010). Three-dimensional structured illumination microscopy has been used to examine the PD by imaging antibody-labeled callose and the VIRAL MOVEMENT PROTEIN (MP) of the tobacco mosaic virus fused to GFP, which interacts with the PD (Fitzgibbon *et al.*, 2010). The simple PD in epidermal cells and central cavities of the complex PD were previously revealed at 100 nm resolution (Roberts and Oparka, 2003; Faulkner *et al.*, 2008). Other than imaging of the molecular complex, observational technology extends to the single molecule level. Internal reflection fluorescence microscopy, stimulated emission depletion, photoactivation localization microscopy, and stochastic optical reconstruction microscopy are examples that may have the potential to observe the PD at the single molecule level (Klar and Hell, 1999; Komis *et al.*, 2015; Schubert, 2017; Paes *et al.*, 2018). However, optimization of the procedures may be necessary when utilized for plasmodesmal imaging, as the plasmodesmal ultrastructure has not yet been revealed by those methods.

## Perspectives

Various plasmodesmal structural forms have been found at different cell–cell interfaces responsible for phloem translocation, including PPUs for phloem loading, large sieve pores for long-distance transport, and funnel PD for unloading. This diverse set of plasmodesmal architectures strongly implies the dynamic function and adaptive ability of PD spatially and temporally. However, several questions remain. Specifically, how do those various forms of PD develop? And, how are the transitions between various architectures regulated? Moreover, other tissues/organs (e.g. fruits) and species (e.g. crops and trees) than Arabidopsis roots have not been investigated, which is likely due to the difficult and time-consuming techniques. It is not known yet if the principal mediation role of PPP for phloem unloading is universal. Due to the developmental variation of vascular tissues, we speculate that more novel plasmodesmal architectures facilitating the adaptation to complex contexts will be discovered. Comparison among plant species would also be helpful for understanding the coevolution between the PD and plants.

The diverse and specific plasmodesmal structures indicate the potential diverse underlying regulatory mechanisms. Concerning the plasmodesmal pore gating system, callose deposition dominated for a long time, until the revelation of the regulation by lipid homeostasis within the plasmodesmal microdomain. Indeed, plasmodesmal structure and permeability are modulated by common factors, such as callose, and also by other unique components. For example, the impact on type I and type II plasmodesmal architectural transition by the *plm* mutation was discovered at the PPP–endodermis interface, but not the SE–PPP interface, which are two successive steps for unloading (Yan *et al.*, 2019). Thus, we believe more unknown regulators will be discovered as direct evidence is revealed. Furthermore, by adjusting the regulation of the plasmodesmal architecture, it is possible to modulate plant development or other biological processes for agricultural production by directing nutrient allocation within plants. Take PLM as an instance: we may be able to change the proportion of plasmodesmal types at specific cell–cell interfaces by altering the sphingolipid levels to modulate symplastic trafficking, enhancing either nutrient unloading for growth promotion or signal molecule movement for stress responses.
